# Associations of Primary Care Provider Burnout with Quality Improvement, Patient Experience Measurement, Clinic Culture, and Job Satisfaction

**DOI:** 10.1007/s11606-024-08633-w

**Published:** 2024-01-25

**Authors:** Denise D. Quigley, Mary Ellen Slaughter, Nabeel Qureshi, Ron D. Hays

**Affiliations:** 1 RAND Corporation, 1776 Main Street, Santa Monica, CA 90401 USA; 2 David Geffen School of Medicine, University of California Los Angeles, Los Angeles, CA USA

**Keywords:** burnout, primary care, quality improvement, job satisfaction

## Abstract

**Background:**

Burnout among providers negatively impacts patient care experiences and safety. Providers at Federally Qualified Health Centers (FQHC) are at high risk for burnout due to high patient volumes; inadequate staffing; and balancing the demands of patients, families, and team members.

**Objective:**

Examine associations of provider burnout with their perspectives on quality improvement (QI), patient experience measurement, clinic culture, and job satisfaction.

**Design:**

We conducted a cross-sectional provider survey about their perspectives including the single-item burnout measure. We fit separate regression models, controlling for provider type, gender, being multilingual, and fixed effects for clinic predicting outcome measures from burnout.

**Participants:**

Seventy-four providers from 44 clinics in large, urban FQHC (52% response rate; *n* = 174).

**Main Measures:**

Survey included a single-item, self-defined burnout measure adapted from the Physician Worklife Survey, and measures from the RAND AMA Study survey, Heath Tracking Physician survey, TransforMed Clinician and Staff Questionnaire, Physician Worklife Survey, Minimizing Errors Maximizing Outcomes survey, and surveys by Friedberg et al. ^31^ and Walling et al. ^32^

**Results:**

Thirty percent of providers reported burnout. Providers in clinics with more facilitative leadership reported not being burned out (compared to those reporting burnout; *p*-values < 0.05). More pressures related to patient care and lower job satisfaction were associated with burnout (*p*-values < 0.05).

**Conclusions:**

Creating provider-team relationships and environments where providers have the time and space necessary to discuss changes to improve care, ideas are shared, leadership supports QI, and QI is monitored and discussed were related to not being burned out. Reducing time pressures and improving support needed for providers to address the high-need levels of FQHC patients can also decrease burnout. Such leadership and support to improving care may be a separate protective factor against burnout. Research is needed to further examine which aspects of leadership drive down burnout and increase provider involvement in change efforts and improving care.

**Supplementary Information:**

The online version contains supplementary material available at 10.1007/s11606-024-08633-w.

## INTRODUCTION

In 2019, the physician burnout rate had for the first time since 2011 dropped below 50% among doctors in the USA, suggesting that healthcare systems were on the right track, but more work needing to be done.^1^ Physician burnout at that time still was much higher than the overall prevalence among US workers of 29% in 2011, and 28% in 2014 and 2017.^2^ The American Medical Association (AMA) began working to mitigate physician burnout and promote professional satisfaction in 2012, commissioning a RAND report in 2013,^3^ convening numerous meetings of experts, healthcare leadership, and other diverse stakeholders (i.e., regulators, payers, EHR vendors), and creating online resources and modules. The growing literature on the prevalence and consequences of burnout in physicians and nurses^4–7^ highlights the need to better address the problem.^8,9^ Physician burnout is significantly associated with poor job satisfaction,^10–12^ decreased productivity,^13^ and lower organizational commitment to wellbeing.^14^ Physician burnout also has a negative impact on patient-reported experience, particularly provider communication.^15,16^ Evidence shows that supportive clinical leadership,^17,18^ sensemaking (organizing data so it is understood well enough to enable reasonable decisions),^19–22^ and sharing of information^3^ may increase provider engagement and morale.

Burnout among providers is particularly salient given the added stressors associated with the COVID-19 pandemic. A recent literature review of provider burnout found the most evidence for workplace, mental health, and psychosocial factors in predicting burnout.^23^ Demographic characteristics were found to have conflicting or no association with burnout. Workplace factors, such as workload, work/life balance, job autonomy, and perceived support from leadership, had strong associations with burnout. Mental health factors, such as anxiety, and physical health risks may increase burnout, but the direction of these associations is unclear because few prospective studies exist.

Although their primary role is to deliver patient care, another key aspect of provider’s jobs is to lead and to facilitate quality improvement (QI).^24^ Providers are often responsible for identifying, assessing, and driving changes in the processes and workflows involved in providing care and interacting with patients and their care. Providers’ role in QI is essential and includes being supportive or engaged as leaders,^25^ supporting a shared understanding of goals, and ensuring alignment of incentives.^26^ Friedberg et al. noted that providers who felt they were providing high-quality care and did not perceive barriers to providing that care had greater professional satisfaction.^3^ Obstacles to providing high-quality care may originate from the practice (e.g., a practice leadership unsupportive of QI ideas) or payers (e.g., payers that refused to cover necessary medical services).

Despite the well-established relationship between provider engagement, physician job satisfaction, supportive leadership, and good information structures with a reduction in provider burnout, there is little evidence about how QI activities relate to provider burnout, and whether QI activities contribute to or mitigate burnout. If QI activities enable providers to digest information and improve the care they provide, it could minimize provider burnout. However, if QI is seen as an added burden, then QI efforts could increase burnout.

To address this gap in the literature, we examine the perspectives of primary care providers about QI, patient experience measurement, clinic culture, and job satisfaction by provider burnout status. We hypothesize the following about burnout among primary care providers:

- **H**_**o**_**1:** Provider “orientation toward and engagement in QI” will be associated with less provider burnout. Specifically, orientation toward and engagement in QI is measured by five items—*QI orientation* (measure [M] 1), *sensemaking* (M2), *concern about reputation* (M3), *desire to improve* (M4), and *worked to improve* (M5)—and we hypothesize that QI orientation, sensemaking, and worked to improve will be associated with less burnout and concern about reputation and desire to improve will be associated with more burnout.
- **H**_**o**_**2:** Higher “engagement in the measurement of patient experience” will be associated with less provider burnout. Specifically, engagement in the measurement of patient experience will be measured by two items—*knowledge of CAHPS performance* (M6) and *CAHPS useful for QI* (M7)—and we hypothesize each is associated with less burnout.
- **H**_**o**_**3:** Positive “clinic culture” will be associated with less provider burnout. Clinic culture is measured by three items—*pressures from patient care* (M8), *facilitative clinic leadership* (M9), and *commitment to clinical outcomes* (M10) and we hypothesize that pressures from patient care will be associated with more burnout, whereas facilitative leadership and commitment to clinical outcomes will be associated with less burnout.
- **H**_**o**_**4:** Provider’s “job satisfaction” will be associated with less provider burnout. Satisfaction with their job is measured by *global job satisfaction* (M11), *satisfaction with individual compensation* (M12), *fairness of P4P incentives* (M13)—and we hypothesize each will be associated with less provider burnout.

## METHODS

### Setting

We partnered with a large, urban Federally Qualified Health Center (FQHC) with 44 primary care clinics in California with nearly 1 million patient visits annually to field this survey. In 2012, the FQHC’s chief medical officer implemented a company-wide quality monitoring system based on the overall provider rating and provider communication composite of the Clinician and Group CAHPS survey (CG-CAHPS) visit survey.

### Data Collection

We developed a survey that asked providers about their clinic culture and experiences as a care provider; it included measures about provider perception of their engagement in and orientation toward QI (QI efforts, desire to improve, resources needed to improvement), patient experience measurement and use of the CAHPS survey (e.g., commitment to use of the survey, thoughts on the usefulness survey, relationship to QI), clinic environment (e.g., care delivery, influence of leadership, support of QI, patient pressures on a clinician), and their thoughts about their job and compensation (e.g., satisfaction with their job, how their compensation is determined, and perceptions of the fairness of their level of compensation). We replicated items from the RAND AMA Study^3^ survey, Heath Tracking Physician Survey,^27^ TransforMed Clinician and Staff Questionnaire (CSQ),^28^ Physician Worklife Survey (PWS),^29^ Minimizing Errors Maximizing Outcomes^30^ (MEMO) provider survey, and Friedberg et al. ^31^ and Walling et al. ^32^ Prior to fielding the survey, we piloted the survey with several practicing primary care providers. We calculated the Cronbach alphas for each composite measure and report these in the results tables.

The survey included 13 established measures (12 multi-item composite measures and 1 single-item measure): perceptions of *QI orientation* (measure [M]1, 6 items), *sensemaking* (M2, 2 items), *concern about reputation* (M3, 2 items), *desire to improve* (M4, 4 items), and *worked to improve* (M5, 7 items), *knowledge of CAHPS performance* (M6, 2 items), *CAHPS useful for QI* (M7, 8 items), *pressures from patient care* (M8, 3 items), *facilitative clinic leadership* (M9, 4 items), *commitment to clinical outcomes* (M10, 1 item), *global job satisfaction* (M11, 2 items), *satisfaction with individual compensation* (M12, 3 items), and *fairness of P4P incentives* (M13, 3 items). Supplemental Table S1 provides the wording of the survey items and response scales.

We also included the single-item self-defined burnout measures adapted from the Physician Worklife Study by Rohland et al. ^33^ The item uses a five-category response scale (scored 1–5): “I enjoy my work. I have no symptoms of burnout.”, “Occasionally I am under stress, and I don’t always have as much energy as I once did, but I don’t feel burned out.”, “I am definitely burning out and have one or more symptoms of burnout, such as physical and emotional exhaustion.”, “The symptoms of burnout that I’m experiencing won’t go away. I think about frustrations at work a lot.”, “I feel completely burned out and often wonder if I can go on. I am at the point where I may need some changes or may need to seek some sort of help.” The item is highly correlated with the full Maslach Burnout Inventory scales and can be used instead of the full inventory.^33^

We administered a web-based survey with email invitations (and 2 follow-up reminders) to active, contracted providers across all clinics within the FQHC from July through August of 2018. Those who completed surveys received a $50 Amazon gift card.

### Data Analysis

Burnout was defined by a response of 3 or higher (see above) to create a dichotomized (0/1) burnout score.

We compared survey respondents (*N* = 74) with the full roster of providers across the clinics within the FQHC (*N* = 143; response rate of 52%) to assess sample representativeness with the overall provider population, using chi-squared tests on the following provider characteristics: specialty, gender, primary language, multilingual status, provider type, role (primary care provider (PCP) or non-PCP), and title (medical doctor, doctor of osteopathy, nurse practitioner, and physician assistant-certified). We did not find any significant differences in provider’s characteristics when comparing respondents with the full population of providers, i.e., no evidence of selection bias.

We compared burnout by provider type, specialty, gender, or being bilingual (i.e., speaking another language). Pearson’s chi-squared tests were used to compare groups by role and specialty, and Fisher’s exact test was used to compare groups by gender and being bilingual.

We had 4 hypotheses including 13 measures (as described in detail above). To test these hypotheses, we fit separate regression models predicting each of the domains using the burnout score as our main independent variable of interest. Initial models controlled for provider type, gender, and being multilingual. We compared the effect of burnout from those models with a model that included indicator variables for the clinic and examined the between- and within-clinic variances for all measures. The final models included the same initial controls and clinic indicators (*n* = 12 clinics). These fixed effects for clinics account for clinic effects such as leadership and QI initiatives. For the main effect in each model, we used an alpha of 0.05 to denote the significance level (i.e., *p*-value < 0.05 the decision rule is to reject the null hypothesis), and due to the study’s exploratory nature, we did not adjust for multiple testing. As a sensitivity analysis, we fit models that also adjusted for the average CG-CAHPS overall provider rating in a 6-month window centered 1 year before the survey because providers’ performance on patient experience measures may influence provider engagement in efforts to improve patient experience. This additional control variable was only available for 85% of respondents.

For each of the study’s provider survey domains that significantly predicted burnout, we also ran models for each of the survey items within the domains. From these models, we calculated adjusted least square means and Cohen’s *d* for effect size. We used the following rule of thumb when interpreting Cohen’s *d*; values 0.2, 0.50, and 0.80 indicate small, medium, and large effect sizes respectively.^34^

All analyses were conducted using R version 3.6.1 including *stat* and *emmeans* packages. Study protocols were approved by our Human Subjects Protection Committee (IRB_Assurance_No: FWA00003425; IRB Number: IRB00000051).

## RESULTS

Thirty percent (22/74) of the providers reported burnout. We found no significant differences between respondents with burnout compared with those not experiencing burnout across provider type, specialty, gender, or speaking another language, or provider’s self-rating of their own communication with patients (Table 1). Half of the providers were physicians (54%), and the majority of providers were women (70%), and not multilingual (77%).

**Table 1:** Primary Care Provider Characteristics and Patient Experience Scores

Characteristics	Not burned out % (*N*)	Burned out + % (*N*)	Overall % (*N*)	*p*-value
	70% (52)	30% (22)	100% (74)	
Provider type				1.000
Physician	54% (28)	55% (12)	54% (40)	
Doctor of osteopathic medicine	7% (2)	25% (3)	13% (5)	
Medical doctor	93% (26)	75% (9)	87% (37)	
Non-physician provider	46% (24)	45% (10)	46% (34)	
Nurse practitioner	83% (20)	80% (8)	82% (28)	
Physician assistant-certified	17% (4)	20% (2)	18% (6)	
Specialty				0.442
Family medicine and internal medicine	59% (31)	77% (17)	65% (48)	
Pediatrics	25% (13)	18% (4)	23% (17)	
Urgent care	10% (5)	5% (1)	8% (6)	
HIV/AIDS	6% (3)	0% (0)	4% (3)	
Gender				1.000
Female	71% (37)	68% (15)	70% (52)	
Male	29% (15)	32% (7)	30% (22)	
Multilingual (yes/no)	21% (11)	27% (6)	23% (17)	0.787
Provider self-rating of communication with their patients +++++ (0–10 scale)	Top box	Top box		
	Percent 9 or 10 (SE)	Percent 9 or 10 (SE)		0.52
	0.43 (0.12)	0.51 (0.09)		
CAHPS patient experience measures	Mean (SE)	Mean (SE)		
Overall provider rating (0–100 score)	90.81 (0.82)	92.25 (1.11)		
Provider communication (0–100 score)	91.11 (0.87)	92.03 (1.18)		

+ burned out versus not burned out is measured by the single, self-defined burnout item, adapted from the Physician Worklife Study, which uses a five-category response scale: 1 = “I enjoy my work. I have no symptoms of burnout.”; 2 = “Occasionally I am under stress, and I don’t always have as much energy as I once did, but I don’t feel burned out.”; 3 = “I am definitely burning out and have one or more symptoms of burnout, such as physical and emotional exhaustion.”; 4 = “The symptoms of burnout that I’m experiencing won’t go away. I think about frustrations at work a lot.”; and 5 = “I feel completely burned out and often wonder if I can go on. I am at the point where I may need some changes or may need to seek some sort of help.”; “Burned out” is defined by a respondent having a score of 3 or higher, resulting in a dichotomized (0/1) burnout scale of burned out and not burned out. +++++ indicates items from the Walling et al. ^32^ study. *CAHPS* stands for Consumer Assessment of Health Care Providers and Systems survey

Three composite domains differed significantly by burnout (Table 2); we report both the domain scores and the items within the domain for only the measures that had significant differences across burnout status; otherwise, we report only the domain score.

**Table 2:**
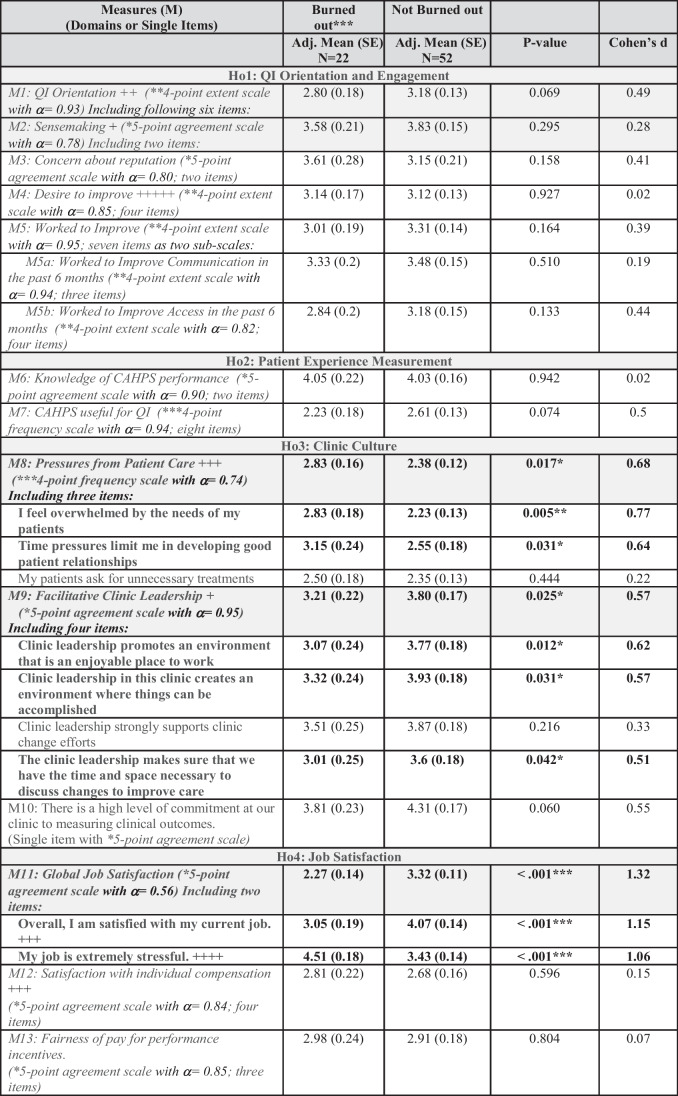
Adjusted Regression Results for Provider Measures Grouped by Hypothesis, By Burned Out vs Not Burned Out

*Italics* indicates a domain of aggregated items (highlighted as light gray rows). **Bold text** indicates statistically significant differences (*p*-value < 0.05) from *t*-tests comparing adjusted means. Items within domains are listed only for statistically significant domains. *The 5-point agreement scale is strongly disagree/somewhat disagree/neither agree or disagree/somewhat agree/strongly agree. **The 4-point extent scale is Not at all, A little, Some, A lot. ***The 4-point frequency scale is Never, Sometimes, Usually, Always. *** indicates burned out versus not burned out is measured by the single, self-defined burnout item, adapted from the Physician Worklife Study, which uses a five-category response scale: 1 = “I enjoy my work. I have no symptoms of burnout.”; 2 = “Occasionally I am under stress, and I don’t always have as much energy as I once did, but I don’t feel burned out.”; 3 = “I am definitely burning out and have one or more symptoms of burnout, such as physical and emotional exhaustion.”; 4 = “The symptoms of burnout that I’m experiencing won’t go away. I think about frustrations at work a lot.”; and 5 = “I feel completely burned out and often wonder if I can go on. I am at the point where I may need some changes or may need to seek some sort of help.”; where “Burned out” is defined by a respondent having a score of 3 or higher, resulting in a dichotomized (0/1) burnout scale of burned out and not burned out. + indicates items from the TransforMed Clinician and Staff Questionnaire (CSQ). ++ indicates items from the Friedberg et al. ^31^ study. +++ indicates items from the Physician Worklife Satisfaction (PWS) survey also measured in the AMA physician survey. ++++ indicates items from the Minimizing Errors Maximizing Outcomes (MEMO) provider survey also measured in the AMA physician survey. +++++ indicates items from the Walling et al^32^ study

We found those who reported having burnout had more *pressures from patient care* (M8: mean difference = 0.45, SE = 0.18, *p*-value = 0.017, Cohen’s *d* = 0.68), less *facilitative* *clinic leadership* (M9: mean difference = −0.59, SE = 0.26, *p*-value 0.025, Cohen’s *d* = 0.57), and significantly lower *global* *job satisfaction* (M11, mean difference = −1.05, SE = 0.16, *p*-value < 0.001, Cohen’s *d* = 1.32). All items within the *pressures* *from patient care* domain had similar associations (higher scores for providers experiencing burnout) and were statistically significant with burnout except for the item *my* *patients ask for unnecessary treatments*. For the *facilitative* *clinic leadership* domain, all items again had a similar association with burnout except for the item *clinic* *leadership strongly supports clinic change efforts*, which was not statistically significant. All items for the *global job satisfaction* domain were significant.

No significant differences were found in burnout for all other measures in our final models that controlled for clinics. In models that did not control for clinics, additional domains were associated with burnout (see Supplemental Table 2). Those reporting burnout had lower QI orientation (M1: mean difference = −0.57, SE = 0.19, *p*-value = 0.004, Cohen’s *d* = 0.73) and lower sensemaking (M2: mean difference = −0.51, SE = 0.23, *p*-value = 0.028, Cohen’s *d* = 0.57), and less commitment at the clinic to measuring clinical outcomes (M10: mean difference = −0.6, SE 0.28, Cohen’s *d* = 0.67). In the sensitivity models that included an additional control for average provider rating in the prior year, the same scales continued to be significant (results not shown).

## DISCUSSION

The largest effect on burnout was found in its relationship with a provider’s global satisfaction with their job. We also identified a relationship between *lower* levels of burnout and more facilitative leadership throughout the clinic as well as providers having fewer pressures from patient care.

About one-third of our sample of primary care providers reported feeling burnout in July and August of 2018. This is roughly the same as the 26% reported for burnout for providers in 2012 pre-pandemic according to the national AMA study that included 447 physicians across 4 states in the USA. Burnout among providers more currently is particularly salient given the added stressors associated with the COVID-19 pandemic; however, the current number of burned-out physicians is much higher than before the pandemic. National studies have shown that in 2023 50% of providers are burned out, compared to 42% in 2018;^35,36^ primary care providers are among the highest who are burned out: family medicine (58%), hospital medicine (59%), and emergency medicine (62%).^11^

Our findings on leadership support felt by providers during change efforts extend previous research conducted on organizational support interventions regarding change.^37^ We found that providers in clinics with leaders that strongly support clinic change efforts ensure that providers have the time and space necessary to discuss changes to improve care, and promote a more enjoyable work environment where things can be accomplished led to less burnout. Actions by leaders to support provider participation in change efforts (e.g., protected time, making space for team building and regular team check-ins) signal to providers the importance of engaging in these efforts which are essential to achieve the desired change outcomes. The organizational behavior literature suggests the value of human connection that comes with QI change efforts. Meaningful projects are essential to support strong teams and the activity of solving problems together adds to a stronger culture.^38^ Also, those reporting more pressures from patient care issues, in terms of time pressure or a high demand from patient needs, were more likely to experience burnout. Lastly, we found providers who reported higher global satisfaction with their jobs were also less burned out.

Additionally, our findings suggest further research on the literature^39^ exploring how QI relates to providers’ morale and engagement; we found suggestive evidence that providers who as teams have the information that they need to do their job well and are more oriented toward and engaged in QI activities at some clinics may be less likely to experience burnout. It appears that in some clinics where physicians are involved in making changes for QI, have teams and staff that share ideas, cooperate in the development and application of new ideas, and are good at making changes and monitoring improvements to the patient care process, the providers are less likely to experience burnout. Also, sensemaking (organizing data so it is understood well enough to enable reasonable decisions) and clinic leadership commitment to measuring outcomes may also be potential mediators of burnout in clinics.

Our study has limitations. We studied one FQHC’s providers’ perceptions, so our findings may not be generalizable. Nonetheless, the study is instructive because we used established measures to inform the limited research on primary care provider work culture, QI, and burnout. We are unable to tease out the direction of causation between QI and burnout among primary care providers; however, the results suggest that facilitative leadership may be a protective factor against burnout. Further study is warranted.

As healthcare systems, including those that care for underserved vulnerable populations, move to more value-based payment structures, they will increasingly rely on providers in FQHCs in their leadership and QI roles to make changes to support patient care and make improvements to care delivery and care experiences. We provide preliminary evidence that creating provider-team relationships and environments where ideas can be shared, leadership supports QI efforts, and QI is monitored and discussed is associated with decreased burnout; one possible interpretation is that such facilitative leadership support for discussing changes and improving care may decrease burnout. Reducing time pressures and improving the support that is needed for providers to address the high-need level of primary care FQHC patients also decrease provider burnout. Common primary care improvement efforts, such as improving open-access scheduling and appointment utilization, may not be specific enough to reduce burnout; research directed at the association of burnout with time spent on doctor-patient interaction centered on care supported by other staff is needed. Our study specifically points to the importance of leaders who strongly support clinic change efforts, ensure that providers have the time and space necessary to discuss changes to improve care, and can promote an enjoyable work environment where things can be accomplished were less likely to experience burnout. Such leadership and support to improve care may be a separate protective factor against burnout. Further inquiry is needed that assesses sensemaking and provider engagement in QI independent from a positive work environment and supportive leadership; research is also needed to examine the direction of the relationship between burnout and which aspects of facilitative leadership improve care by promoting provider involvement in change efforts and reducing burnout.

### Supplementary Information

Below is the link to the electronic supplementary material.

Supplementary file1 (DOCX 30.2 KB)

## Data Availability

The datasets during and/or analyzed during the current study are available from the corresponding author on reasonable request.
