# Supplementary material for: Associations of Primary Care Provider Burnout with Quality Improvement, Patient Experience Measurement, Clinic Culture, and Job Satisfaction
Source: J Gen Intern Med. 2024 Jan 25;39(9):1567–74. doi: 10.1007/s11606-024-08633-w (PMC11255139; doi:10.1007/s11606-024-08633-w)
Supplement: Supplementary file 1 — Supplementary file1 (DOCX 30.2 KB) [file 11606_2024_8633_MOESM1_ESM.docx]

**Supplemental Table S1. Provider Survey Measures and their Items Grouped By Hypothesis**

| **Measures (M)** | |
| --- | --- |
| **Domains (and their items) or Single Items** | **Response scale** |
| **Ho1: Quality Improvement (QI) Orientation and Engagement** | |
| ***M1: QI Orientation ++***  To what extent has your clinic done the following to support quality improvement in the past 6 months? | 4-point extent scale:  Not at all/A little/Some/A lot |
| Involved support staff in making changes for QI |  |
| Involved physicians in making changes for QI |  |
| People in our clinic cooperate to develop and apply new ideas |  |
| Our clinic is good at changing care processes to make sure problems don’t recur |  |
| After our clinic makes changes to improve the patient care process, we check to see if the changes worked |  |
| Our clinic encourages everyone to share ideas |  |
| ***M2: Sensemaking***  Please indicate how much you agree or disagree with the following statements about your clinic. | 5-point agreement scale:  strongly disagree/somewhat disagree/neither agree or disagree/ somewhat agree/strongly agree |
| When we experience a problem in the clinic, we make a serious effort to figure out what’s really going |  |
| People in this clinic have the information that they need to do their jobs well |  |
| ***M3: Concern about reputation***  Based on your experiences in the last 6 months, how much do you agree or disagree with the following statements. | 5-point agreement scale:  strongly disagree/somewhat disagree/neither agree or disagree/ somewhat agree/strongly agree |
| I am concerned about my individual reputation among patients because of CAHPS survey data |  |
| I am concerned about my individual reputation with clinic leadership (e.g., medical director, site clinic administrator) because of CAHPS survey data |  |
| ***M4:* *Desire to improve* ++++**  Please indicate how much you want to improve ... | 4-point extent scale:  Not at all/A little/Some/A lot |
| Your ability to communicate with patients |  |
| Your ability to communicate with other members of your care team |  |
| Your ability to coordinate information external to the practice to support patient care (e.g., referrals, inpatient stays, etc.) |  |
| Your patients' overall ratings of you on CAHPS surveys |  |
| ***M5: Worked to Improve***  In the last 6 months, did you work to improve your own behaviors and interactions with patients in the following areas? | 4-point extent scale:  Not at all/A little/Some/A lot |
| Communicating directly with patients during office visits |  |
| Providing information about health questions or concerns that is easier for patients to understand |  |
| Showing respect for what patients have to say |  |
| Responding to patients who contact the office with medical questions more quickly |  |
| Scheduling timely appointments for non-urgent care |  |
| Scheduling timely appointments for urgent care |  |
| Seeing patients within 15 minutes of their appointment time more consistently |  |
| **Ho2: Patient Experience Measurement** | |
| ***M6: Knowledge of CAHPS performance***  Based on your experiences in the last 6 months, how much do you agree or disagree with the following statements. | 5-point agreement scale:  strongly disagree/somewhat disagree/neither agree or disagree/ somewhat agree/strongly agree |
| Patients’ written comments (about a good or bad experience) have been an important source of quality improvement ideas for me |  |
| CAHPS survey data have motivated me to make changes in how I deliver care |  |
| ***M7: CAHPS useful for QI***  Based on your experiences in the last 6 months, how often have the following been true. | 4-point frequency scale:  Never/Sometimes/Usually/Always |
| CAHPS survey data has provided useful information about how well our clinic runs |  |
| CAHPS survey data has provided useful information about my own performance |  |
| CAHPS survey data have been an important source of quality improvement ideas for me |  |
| Patients’ written comments (about a good or bad experience) have been an important source of quality improvement ideas for me |  |
| CAHPS survey data have motivated me to make changes in how I deliver care |  |
| Patients’ written comments (about a good or bad experience) have motivated me to make changes in how I deliver care |  |
| I have made changes to my care team based on CAHPS survey data |  |
| I have made changes to my own behavior and interaction with patients based on CAHPS survey data |  |
| **Ho3: Clinic Culture** | |
| ***M8: Pressures from Patient Care +++***  Based on your experiences in the last 6 months, how often have the following been true. | 4-point frequency scale:  Never/Sometimes/Usually/Always |
| I feel overwhelmed by the needs of my patients |  |
| Time pressures limit me in developing good patient relationships |  |
| My patients ask for unnecessary treatments |  |
| ***M9: Facilitative Clinic Leadership* +**  Please indicate how much you agree or disagree with the following statements about your clinic. | 5-point agreement scale:  strongly disagree/somewhat disagree/neither agree or disagree/ somewhat agree/strongly agree |
| Clinic leadership promotes an environment that is an enjoyable place to work |  |
| Clinic leadership in this clinic creates an environment where things can be accomplished |  |
| Clinic leadership strongly supports clinic change efforts |  |
| The clinic leadership makes sure that we have the time and space necessary to discuss changes to improve care |  |
| **M10: Commitment at clinic to measuring clinical outcomes**  Based on your experiences in the last 6 months, how much do you agree or disagree with the following statements.  There is a high level of commitment at our clinic to measuring clinical outcomes. | 5-point agreement scale:  strongly disagree/somewhat disagree/neither agree or disagree/ somewhat agree/strongly agree |
| **Ho4: Job Satisfaction** | |
| ***M11: Global Job Satisfaction***  Please indicate how much you agree or disagree with the following statements. | 5-point agreement scale:  strongly disagree/somewhat disagree/neither agree or disagree/ somewhat agree/strongly agree |
| Overall, I am satisfied with my current job |  |
| My job is extremely stressful |  |
| ***M12: Satisfaction with individual compensation* +++**  Please indicate how much you agree or disagree with the following statements. | 5-point agreement scale:  strongly disagree/somewhat disagree/neither agree or disagree/ somewhat agree/strongly agree |
| My total compensation package is fair |  |
| I am not well compensated given my training and experience |  |
| I am not well compensated compared to physicians in other specialties |  |
| I am not well compensated compared to other physicians in my practice |  |
| ***M13: Fairness of pay for performance incentives***  Please indicate how much you agree or disagree with the following statements. | 5-point agreement scale:  strongly disagree/somewhat disagree/neither agree or disagree/ somewhat agree/strongly agree |
| The methods used to calculate my bonus every 6 months are fair |  |
| The amount of the bonus received based on my individual physician performance is fair |  |
| Including measures of patient reported experiences from CAHPS surveys as part of my performance is fair |  |

NOTE: *Italics* indicates a domain of aggregated items. + indicates items from the TransforMed Clinician and Staff Questionnaire (CSQ). ++ indicates items from the Friedberg et al. 2016 study. +++ indicates items from the Physician Worklife Satisfaction (PWS) survey also measured in the AMA physician survey. ++++ indicates items from the Minimizing Errors Maximizing Outcomes (MEMO) provider survey also measured in the AMA physician survey. +++++ indicates items from the Walling et al., 2009 study.

**Supplemental Table S2. Adjusted Regression Results without clinic controls for Provider Measures Grouped by Hypothesis, By Burned Out vs Not Burned Out**

| **Measures (M)**  **(Domains or Single Items)** | **Burned out***** | **Not Burned out** |  |  |
| --- | --- | --- | --- | --- |
|  | **Adj. Mean (SE)**  **N=22** | **Adj. Mean (SE)**  **N=52** | **P-value** | **Cohen’s d** |
| **Ho1: QI Orientation and Engagement** | | | | |
| ***M1: QI Orientation ++ (**4-point extent scale with α= 0.93) Including following six items:*** | **2.64 (0.17)** | **3.21 (0.13)** | **0.004**** | **0.73** |
| Involved support staff in making changes for QI | 2.81 (0.19) | 3.09 (0.14) | 0.185 | 0.34 |
| **Involved physicians in making changes for QI** | **2.19 (0.23)** | **2.89 (0.17)** | **0.008**** | **0.66** |
| **People in our clinic cooperate to develop and apply new ideas** | **2.57 (0.2)** | **3.13 (0.15)** | **0.016*** | **0.62** |
| **Our clinic is good at changing care processes to make sure problems don’t recur** | **2.33 (0.2)** | **3.10 (0.15)** | **0.001**** | **0.85** |
| **After our clinic makes changes to improve the patient care process, we check to see if the changes worked** | **2.29 (0.21)** | **2.92 (0.16)** | **0.010*** | **0.66** |
| **Our clinic encourages everyone to share ideas** | **2.55 (0.23)** | **3.09 (0.17)** | **0.033*** | **0.55** |
| ***M2: Sensemaking* + *(*5-point agreement scale with α= 0.78) Including two items:*** | **3.35 (0.21)** | **3.86 (0.15)** | **0.028*** | **0.57** |
| When we experience a problem in the clinic, we make a serious effort to figure out what’s really going on | 3.35 (0.25) | 3.80 (0.19) | 0.113 | 0.43 |
| **People in this clinic have the information that they need to do their jobs well** | **3.37 (0.21)** | **3.89 (0.16)** | **0.030*** | **0.57** |
| *M3: Concern about reputation (*5-point agreement scale with α= 0.80; two items)* | 3.50 (0.26) | 3.21 (0.20) | 0.325 | 0.26 |
| *M4:* *Desire to improve* +++++ *(**4-point extent scale with α= 0.85; four items)* | 3.03 (0.17) | 3.13 (0.13) | 0.612 | 0.13 |
| *M5: Worked to Improve (**4-point extent scale with α= 0.95; seven items as two sub-scales:* | 3.01 (0.18) | 3.31 (0.13) | 0.122 | 0.40 |
| *M5a: Worked to Improve Communication in past 6 mos (**4-point extent scale with α= 0.94; three items)* | 3.30 (0.19) | 3.48 (0.14) | 0.391 | 0.23 |
| *M5b: Worked to Improve Access in past 6 months (**4-point extent scale with α= 0.82; four items)* | 2.86 (0.18) | 3.19 (0.14) | 0.115 | 0.41 |
| **Ho2: Patient Experience Measurement** | | | | |
| *M6: Knowledge of CAHPS performance (*5-point agreement scale with α= 0.90; two items)* | 3.90 (0.21) | 3.98 (0.16) | 0.752 | 0.08 |
| *M7: CAHPS useful for QI (***4-point frequency scale with α= 0.94; eight items)* | 2.22 (0.17) | 2.54 (0.13) | 0.092 | 0.43 |
| **Ho3: Clinic Culture** | | | | |
| ***M8: Pressures from Patient Care +++***  ***(***4-point frequency scale with α= 0.74) Including three items:*** | **2.86 (0.15)** | **2.38 (0.11)** | **0.004**** | **0.73** |
| **I feel overwhelmed by the needs of my patients** | **2.88 (0.17)** | **2.24 (0.13)** | **0.001**** | **0.81** |
| **Time pressures limit me in developing good patient relationships** | **3.22 (0.21)** | **2.52 (0.16)** | **0.003**** | **0.74** |
| My patients ask for unnecessary treatments | 2.49 (0.17) | 2.38 (0.13) | 0.537 | 0.16 |
| ***M9: Facilitative Clinic Leadership* +  *(*5-point agreement scale with α= 0.95) Including four items:*** | **3.02 (0.23)** | **3.86 (0.17)** | **0.001**** | **0.81** |
| **Clinic leadership promotes an environment that is an enjoyable place to work** | **2.87 (0.24)** | **3.84 (0.18)** | **0.001**** | **0.86** |
| **Clinic leadership in this clinic creates an environment where things can be accomplished** | **3.14 (0.24)** | **3.98 (0.18)** | **0.002**** | **0.78** |
| **Clinic leadership strongly supports clinic change efforts** | **3.27 (0.25)** | **3.91 (0.19)** | **0.021*** | **0.59** |
| **The clinic leadership makes sure that we have the**  **time and space necessary to discuss changes to improve care** | **2.83 (0.26)** | **3.68 (0.19)** | **0.004**** | **0.74** |
| **M10: There is a high level of commitment at our clinic to measuring clinical outcomes.**  **(Single item with **5-point agreement scale)*** | **3.72 (0.21)** | **4.32 (0.16)** | **0.010*** | **0.67** |
| **Ho4: Job Satisfaction** | | | | |
| ***M11: Global Job Satisfaction (*5-point agreement scale with α= 0.56) Including two items:*** | **2.28 (0.14)** | **3.39 (0.11)** | **< .001***** | **1.38** |
| **Overall, I am satisfied with my current job. +++** | **3.00 (0.17)** | **4.07 (0.13)** | **< .001***** | **1.22** |
| **My job is extremely stressful. ++++** | **4.43 (0.20)** | **3.30 (0.15)** | **< .001***** | **1.10** |
| *M12: Satisfaction with individual compensation* +++  *(*5-point agreement scale with α= 0.84; four items)* | 2.70 (0.21) | 2.78 (0.16) | 0.724 | 0.09 |
| *M13: Fairness of pay for performance incentives.*  *(*5-point agreement scale with α= 0.85; three items)* | 2.92 (0.21) | 2.91 (0.16) | 0.940 | 0.02 |

NOTE: *Italics* indicates a domain of aggregated items (highlighted as light grey rows). **Bold** text indicates statistically significant differences (p-value<0.05) from t-tests comparing adjusted means. Items within domains are listed only for statistically significant domains. *The 5-point agreement scale is: strongly disagree/somewhat disagree/neither agree or disagree/ somewhat agree/strongly agree. **The 4-point extent scale is Not at all, A little, Some, A lot. ***The 4-point frequency scale is Never, Sometimes, Usually, Always. ***** indicates Burned out versus Not Burned out is measured by the single, self-defined burnout item, adapted from the Physician Worklife Study,, which uses a five-category response scale: 1 = “I enjoy my work. I have no symptoms of burnout.”; 2 = “Occasionally I am under stress, and I don’t always have as much energy as I once did, but I don’t feel burned out.”; 3 = “I am definitely burning out and have one or more symptoms of burnout, such as physical and emotional exhaustion.”; 4 = “The symptoms of burnout that I’m experiencing won’t go away. I think about frustrations at work a lot.”; and 5 = “I feel completely burned out and often wonder if I can go on. I am at the point where I may need some changes or may need to seek some sort of help.”; where “Burned out” is defined by a respondent having a score of 3 or higher, resulting in a dichotomized (0/1) burnout scale of Burned out and Not Burned out.+ indicates items from the TransforMed Clinician and Staff Questionnaire (CSQ). ++ indicates items from the Friedberg et al. 2016 study. +++ indicates items from the Physician Worklife Satisfaction (PWS) survey also measured in the AMA physician survey. ++++ indicates items from the Minimizing Errors Maximizing Outcomes (MEMO) provider survey also measured in the AMA physician survey. +++++ indicates items from the Walling et al., 2009 study.
